# INTRAINDIVIDUAL VARIABILITY ACROSS ADULTHOOD: DIVERSITY, VARIETY, AND ADHERENCE AS KEY INDICATORS OF DAILY LIFE

**DOI:** 10.1093/geroni/igad104.0922

**Published:** 2023-12-21

**Authors:** Rachel Koffer, Soomi Lee, Susan Charles

## Abstract

Alongside long-term change, lifespan theories highlight the importance of examining intraindividual variability (the fluctuations that occur in daily and momentary life) as an indicator of dynamic characteristics, like plasticity or rigidity. The present symposium demonstrates five unique characterizations and implications of variability in daily life across the adult lifespan. Paper 1 demonstrates that greater social activity variety is linked to lower mortality risk over 11 years, and this effect is stronger for older adults. Paper 2 finds that consistent adherence to health behaviors in daily life is linked to better cognitive function in midlife adults. Paper 3 finds that middle-aged adults experience greater diversity in types of positive events in daily life (positive event diversity) compared to older adults, and positive event diversity is associated with higher positive affect. Paper 4 establishes that positive affect with high mean levels and high variability (fragile high positive affect) is associated with greater odds of depression 10 years later. Lastly, Paper 5 demonstrates that although the spread of stressor types experienced in daily life (stressor diversity) declines with age across both cohorts, the types of stressors experienced in modern life differ from those experienced 18 years prior. Together, findings demonstrate how variability in daily life changes across age and has implications for health and well-being of older adults. Dr. Susan Charles will critically discuss these contributions to our understanding of aging in the context of Strength and Vulnerability Integration Theory and provide considerations for understanding the complexity of daily life across the lifespan.

